# Identification of genomic regions associated with agronomic and biofortification traits in DH populations of rice

**DOI:** 10.1371/journal.pone.0201756

**Published:** 2018-08-10

**Authors:** B. P. Mallikarjuna Swamy, Gwen Iris L. Descalsota, Chau Thanh Nha, Amery Amparado, Mary Ann Inabangan-Asilo, Christine Manito, Frances Tesoro, Russell Reinke

**Affiliations:** 1 Plant Breeding Platform, International Rice Research Institute (IRRI), Metro Manila, Philippines; 2 University of Southern Mindanao, Kabacan, Cotabato, Philippines; 3 Cuu Long Delta Rice Research Institute (CLRRI), Can Tho, Vietnam; Huazhong University of Science and Technology, CHINA

## Abstract

Rice provides energy and nutrition to more than half of the world’s population. Breeding rice varieties with the increased levels of bioavailable micronutrients is one of the most sustainable approaches to tackle micronutrient malnutrition. So, high zinc and iron content in the grain are primary targets in rice biofortification breeding. In this study, we conducted QTL mapping using doubled haploid (DH) populations, PSBRc82 x Joryeongbyeo and PSBRc82 x IR69428, phenotyped for agronomic traits and micronutrients during two growing seasons and using genotypic information from analysis with the 6K SNP chip. A number of DH lines were identified as having high grain Zn and Fe content in polished rice. Importantly, we identified 20 QTLs for agronomic traits and 59 QTLs for a number of biofortification traits. Of the 79 QTLs, 12 were large-effect QTLs (>25% PVE), nine QTLs were consistent across seasons in either population, and one QTL was identified in both populations. Moreover, at least two QTLs were clustered in defined regions of chromosomes 1, 2, 3, 4, 5, 7 and 9. Eight epistatic interactions were detected for Cu, Mg, Na, and Zn in population 1. Furthermore, we identified several candidate genes near QTLs for grain Zn (*OsNRAMP*, *OsNAS*, *OsZIP*, *OsYSL*, *OsFER*, and *OsZIFL* family) and grain yield (*OsSPL14* and *OsSPL16*). These new QTLs and candidate genes help to further elucidate the genetic basis for grain micronutrient concentration, and may prove useful for marker assisted breeding for this important trait.

## Introduction

Rice (*Oryza sativa* L.) is one of the most vital food plants in the world. It provides energy and nutrition to nearly half of the people on earth [1]. In most of the developing countries in Asia, rice is eaten in significant quantities almost every day and it is the major component of the daily diet of the population since there are limited opportunities and resources to diversify the diet with fruits, vegetables, and meat [2]. Further, modern high-yielding rice varieties are low in mineral elements with polished rice having even lower amounts. Thus, milled rice is not a major supplier of any of the mineral elements in significant quantities and cannot meet the recommended daily dietary intake [3]. Consequently, most rice-eating, resource-poor people in South and Southeast Asia, Africa, and Latin America suffer from chronic micronutrient malnutrition, often called hidden hunger [4].

Mineral elements, such as iron (Fe), zinc (Zn), calcium (Ca), magnesium (Mg), potassium (K), manganese (Mn), boron (B), phosphorus (P), and copper (Cu), are essential for many cellular and metabolic functions and important structural components of many tissues, fluids and vital organs. So, they are highly beneficial for the growth and development of plants and animals [5]. However, some elements, such as arsenic (As) and cadmium (Cd), have detrimental effects on humans [6]. Arsenic causes cancer, neurological disorders, and circulatory diseases; Cd causes renal and liver disorders, weak bones, anemia, and hypertension [7]. Thus, the concentrations of these mineral elements in staple foods, such as rice, have a huge implication on human health. In addition, the potential contamination of rice with toxic elements may also have a huge impact on the rice trade [8].

Among different mineral elements, Fe and Zn are essential for human health. Zn is a major cofactor for several vital enzymes and Fe is an important component of blood. Deficiency of these elements in the diet results serious human health problems, especially among children and pregnant and lactating women [9]. Zn deficiency causes stunting, diarrhea, and reduced immunity, while Fe deficiency causes anemia. Approximately 2 billion people suffer from Fe and Zn deficiencies globally, and it is a high priority to address these deficiencies in order to achieve the sustainable development goals such as reduced child and maternal mortalities and a reduction in poverty and hunger [10]. Biofortification of staple crops has emerged as a sustainable strategy to overcome mineral deficiencies. Using this approach, CGIAR centers in collaboration with HarvestPlus have made significant progress in developing, releasing, and disseminating biofortified crops [4].

The accumulation of mineral elements in the grain is a complex process and highly influenced by environmental factors. This made early-generation phenotypic-based selections for grain mineral elements, particularly grain Zn less effective and has slowed progress in breeding for biofortified rice varieties [11]. A thorough understanding of the genetic basis of grain mineral elements at the molecular level and identifying major-effect QTLs can assist in faster development of biofortified rice varieties through marker-assisted breeding (MAB) [12].

As rice has a relatively small genome and has long been the focus of significant genetic research as a model for the cereal crops, there are enormous genomic resources available, including genome-wide single-nucleotide polymorphic (SNP) molecular markers and various advanced genomic platforms, to allow dissection of complex traits at the molecular level [13]. Some of the recent efforts to map QTLs for mineral nutrients include the use of introgression lines (ILs) for Mn, Ca, Cu, Mg, P, K, Fe, and Zn [3] and DHs to discover QTLs for P, Cu, Mn, Fe, and Zn [14]. Several major-effect epistatic loci were reported for Fe and Zn [15]. Twenty-three and nine QTLs, respectively, were identified in two environments for grain concentrations of Ca, Fe, K, Mg, Mn, P, and Zn [16]. Using RILs, 14 QTLs for grain Fe and Zn were identified and prioritized the candidate genes underlying these QTLs and known to be involved in metal homeostasis: *OsYSL1* and *OsMTP1* for Fe; *OsNAS1* and *OsNAS2* for Zn; and *OsNAS3*, *OsNRAMP1*, and *APRT* for both Fe and Zn [17]. Similarly, several metal homeostasis genes validated in a RIL population shown significant association with grain Zn content [18]. Meanwhile, QTLs were mapped for 19 mineral elements under multiple environments [12]. All these studies resulted in identification of multiple loci distributed throughout the genome with low to moderate genetic effects [12]. The majority of these loci were specific to different genetic backgrounds and environments and seldom used in MAB.

Among different biparental populations, DHs are fixed genetic materials that can be developed faster relative to other mapping populations, can be evaluated across years and locations readily, and have less genetic background ‘noise’ that make them important genetic resources for mapping QTLs/genes for various traits [19]. Several reports have shown the utility of DH populations in identifying QTLs for the concentration of elements in grain [14] [20].

The main objectives of our study were to evaluate doubled haploid mapping populations for yield, yield components, and grain micronutrient traits; map QTLs for yield and grain micronutrients; understand the QTL x QTL interactions; identify candidate genes associated with major effect Zn QTLs; compare QTLs for grain micronutrients with earlier QTLs; and identify promising lines with high grain Zn and yield.

## Materials and methods

### Experimental location and plant materials

These studies were conducted during the 2015DS (dry season) and 2015WS (wet season) at the International Rice Research Institute (IRRI), Philippines. We used two DH populations consisting, respectively, of 130 and 97 lines derived from PSBRc82 x Joryeongbyeo (P1) and PSBRc82 x IR69428 (P2). The PSBRc82 is one of the most widely adapted and popular rice varieties in the Philippines, while Joryeongbyeo is a Korean rice variety found to have high Zn content and IR69428 is a breeding line (IR 65564-44-5-2/SENGKEU//IR 65600-1-3-2) having high Zn content.

### Phenotypic evaluation of mapping populations

The experiments were carried out following a randomized complete block design (RCBD) with three replications; with check varieties used for yield and micronutrient comparisons. The populations were phenotyped for four agronomic traits and 13 grain micronutrient traits. The agronomic traits were measured following the standard evaluation system [21], including days to 50% flowering (DTF; number of days taken from sowing to the time that 50% of plants of the family showed flowering), plant height (PH; height from the soil surface to the tip of the primary panicle identified at the time of harvest), number of tillers (NT; average number of tillers from three plants at the time of harvest) and yield per hectare (YLD; average weight of the cleaned grains dried to 14% moisture from all the plants per plot). For mineral analysis of the grain, 50 g of paddy samples were dehulled using a Satake dehuller and milled for one minute using a K-710 mini-lab rice polisher. Milled rice samples weighing at least 3g representing each plot were analyzed using X-ray Fluorescence Spectrometry (XRF) (Oxford) to measure Fe and Zn [22]. While, brown rice samples from two replications were analyzed for all the biofortification elements using Inductively Coupled Plasma Mass Spectrometry (ICP-MS) at Flinders University, Australia. The average reading per plot was used for subsequent statistical analysis.

### Genotyping

We collected leaf samples from 227 DH lines and parental lines, which were ground after freezing with liquid nitrogen. The genomic DNA was extracted using modified CTAB protocol [23]. The DNA quality was checked by 1.5% of Agarose gel electrophoresis. High-quality DNA samples with appropriate concentrations (~50 ng) were submitted for 6K SNP Infinium Assay. Scanned image calls and automatic allele calling were loaded in the Illumina Genome Studio data analysis version V2010.1.

### Statistical analysis

Descriptive statistics were generated using STAR v.2.0.1, while Analysis of Variance (ANOVA) was carried out using PBTools v1.4. Histograms and correlations between pairs of traits were estimated through Pearson correlation co-efficients using R software. The model used for ANOVA was:

$$Y_{ijk}=\mu +\alpha_{i}+r_{j}+b_{kj}+\varepsilon_{ijk}$$

where *μ* is the overall mean, *α*_*i*_ is the effect of the *i*^*th*^ genotype; *r*_*j*_ is the effect of the *j*^*th*^ replicate, *b*_*kj*_ the effect of the *k*^*th*^ block within the *j*^*th*^ replicate and *ε*_*ijk*_ the error. The genotypes were considered fixed while replicates and blocks within replicates were random.

For estimating broad sense heritability, variance components were estimated considering all factors including genotypes as random. For each group, broad-sense heritability or repeatability (H^2^) for each season was calculated as:

$$H^{2}=\frac{\sigma_{g}^{2}}{\sigma_{p}^{2}}\;and\;\sigma_{p}^{2}=\sigma_{g}^{2}+\frac{\sigma_{e}^{2}}{r}$$

Where $\sigma_{p}^{2}$ is the phenotypic variance, $\sigma_{g}^{2}$ is the genotypic variance, $\sigma_{e}^{2}$is the error variance and r is the number of replications in the season.

#### Linkage mapping and QTL analysis

Marker data sets generated from 6K SNP genotyping were screened based on >80% call rate, homozygosity, and polymorphism between respective parents. Scoring of alleles in DH populations was adjusted at each SNP locus through comparing parental alleles at the respective SNP locus as dominant markers. The maternal alleles were scored as ‘A’, paternal alleles as ‘B’. Ambiguous or missing data were shown as “X”. Redundant SNP markers (i.e., completely correlated markers) were removed and the remaining SNP markers were anchored, grouped, and ordered by the input. Moreover, SNP markers that were considered unlinked were removed from the linkage map in order to attain the appropriate linkage map lengths. Linkage maps of the two DH populations were created following Kosambi function using IciMapping ver.4.1 [24]. QTLs were identified by inclusive composite interval mapping (ICIM) in QTL IciMapping ver.4.1. The average trait values for each line in each DH population were used for the QTL analysis. QTLs were named following the standard procedures. The LOD thresholds of the QTL were set based on 1,000-permutation test at a 95% confidence level. The proportion of observed phenotypic variance explained by each QTL and the corresponding additive effects were also estimated. The digenic (epistatic) interactions between marker loci were determined by setting the LOD threshold of 6.0. QTLs were visualized using MapChart v.2.3 [25].

### QTL search and candidate gene analysis

Annotated genes with functions related to metal homeostasis, loading, sequestration, and transport of mineral elements were compiled. The physical positions of these annotated genes were determined using the RAP-DB Genome Browser (http://rapdb.dna.affrc.go.jp/viewer/gbrowse/irgsp1). Annotation and functions attributed to different candidate genes were downloaded from *Oryzabase* (https://shigen.nig.ac.jp/rice/oryzabase/gene) and RiceChip (www.ricechip.org). Metal transport and heavy metal homeostasis genes physically located within or near QTLs for agronomic and biofortification traits were considered candidate genes.

## Results

### Phenotypic analysis

All the agronomic and biofortification traits showed typical normal distributions (Table 1, S1 and S2 Figs). The analysis of variance showed that there was significant genotypic variation for all the traits in the two populations during the DS and WS. The mean, range, standard derivation (SD), co-efficient of variation (CV), and heritability (H^2^) values are presented in Table 1. The highest mean values for PH, DTF, and NT were recorded in WS, whereas the highest value of YLD was observed during DS. The general trend of biofortification traits was that higher values were recorded during DS except for grain Fe and Zn. The CV was lower than 10% for DTF, K, Mg, and P in P1 and for Mg, and P in P2, whereas CVs ranged from moderate to high (10.38%–52.61%) for all the other traits. Broad sense heritability was high (60%–97%) for all the traits in both the seasons and populations, except for Fe and NT.

**Table 1:** Variability for agronomic and biofortification traits in P1 and P2 during 2015WS and 2015DS.

Trait	Range		Mean ± SD		CV (%)		F-value		H^2^		Pop
	DS	WS	DS	WS	DS	WS	DS	WS	DS	WS	
**DTF**	75–99	72–98	82.37±0.39	87.98±0.51	5.92	7.29	8.95	11.23	0.89	0.91	1
	75–105	70–109	87.67±0.92	88.3±0.88	10.48	9.97	31.4	39.93	0.97	0.97	2
**PH**	57–119	60–152	80.46±0.89	84.98±1.09	13.81	17.27	11.26	6.99	0.91	0.86	1
	65–113	61–114	90.24±1.02	93.32±1.1	11.97	12.9	9.06	7.79	0.89	0.87	2
**NT**	11–29	11–28	15.95±0.29	19.38±0.32	29.08	27.59	2.1	1.95	0.52	0.49	1
	11–24	12–30	15.53±0.27	17.45±0.32	25.67	26.19	1.67	1.81	0.4	0.45	2
**YLD**	1449–8022	854–3985	4720.8±99.2	2205.6±63.45	31.56	41.69	2.7	4.05	0.63	0.75	1
	2489–7462	580–5295	5082.3±115.25	2833.4±106.2	28.78	41.63	3.05	5.72	0.67	0.83	2
**As**	0.12–0.28	0.13–0.33	0.21±0.004	0.2±0.003	27.63	22.34	3.35	6.36	0.7	0.84	1
	0.13–0.40	0.16–0.38	0.26±0.01	0.26±0.005	24.08	20.69	9.14	6.14	0.88	0.84	2
**B**	6.70–15.30	2.40–6.20	10.33±0.19	4.34±0.06	22.86	20.06	4	4.23	0.75	0.76	1
	8.90–22.00	3.30–6.90	13.17±0.27	4.99±0.08	21.31	18.45	5.51	5.39	0.8	0.82	2
**Ca**	57–129	53–121	90.45±1.56	82.62±1.3	18.56	19.24	12.6	14.11	0.92	0.93	1
	66–156	58–111	93.15±1.73	81.82±1.2	17.33	15.33	13.09	12.73	0.91	0.92	2
**Co**	0.01–0.04	0.02–0.07	0.03±0.0006	0.04±0.0009	23.58	28.27	8.09	4.13	0.88	0.76	1
	0.02–0.05	0.02–0.08	0.03±0.0007	0.04±0.001	22.18	34.73	6.92	6.46	0.84	0.84	2
**Cu**	3.00–5.60	2.00–5.20	4.11±0.05	3.3±0.06	16.65	21.7	3.59	7.49	0.72	0.87	1
	2.70–7.00	2.40–6.70	4.29±0.09	3.71±0.08	19.84	22.46	7.11	12.98	0.85	0.92	2
**Fe**	1.18–2.52	1.88–7.87	1.78±0.03	4.65±0.09	23.94	31.14	1.54	1.77	0.34	0.44	1
	1.20–2.63	3.27–6.83	1.71±0.03	4.78±0.08	23.82	26.94	1.42	1.12	0.31	0.11	2
**K**	2933–4233	2867–4167	3496±27.66	3399.2±22.7	9.04	9.34	6.07	4.23	0.84	0.76	1
	2600–4133	2533–3800	3323.3±38.43	3090.8±30.82	10.38	10.86	13.62	7.66	0.92	0.87	2
**Mg**	1370–1940	1207–1707	1595.2±11.83	1425.4±8.04	8.24	7.76	8.55	4.66	0.88	0.79	1
	1237–1690	1137–1653	1444±11.81	1343.2±9.14	7.71	7.65	6.93	5.58	0.84	0.82	2
**Mn**	20–39	19–39	26.34±0.44	25.96±0.4	18.54	18.82	6.94	14.93	0.86	0.93	1
	17–40	17–33	26.44±0.56	23.72±0.38	21.07	17	7.44	12.57	0.85	0.92	2
**Mo**	0.50–3.60	0.28–2.89	1.73±0.07	1.16±0.05	43.53	47.42	15.99	28.16	0.94	0.96	1
	0.40–2.30	0.30–1.90	1.17±0.07	0.94±0.05	49.12	52.61	26.18	15.76	0.96	0.94	2
**Na**	9–45	7–38	19.44±0.77	15.78±0.54	45.68	46.91	5.89	4.96	0.83	0.8	1
	6.30–33.3	8–30	16.87±0.71	13.02±0.39	39.01	37.85	9.68	2.87	0.89	0.65	2
**P**	3267–4950	3367–4967	3959.6±36.65	3958.2±25.38	9.99	8.9	10.92	4.38	0.91	0.77	1
	3000–4600	3133–4500	3650.19±37.66	3611.4±29.42	9.51	8.98	9.33	6.72	0.88	0.85	2
**Zn**	11.28–26.35	13.17–24.8	16.65±0.24	17.57±0.21	17.98	16.69	8.27	4.43	0.88	0.77	1
	9.95–22.78	12.33–23.43	16.44±0.24	17.11±0.24	16.81	16.94	4.09	3.75	0.76	0.73	2

SD: standard deviation; CV: co-efficient of variation; H^2^: heritability; DTF: days to 50% flowering; PH: plant height; NT: number of tillers; YLD: grain yield; As: arsenic; B: boron; Ca: calcium; Co: cobalt; Cu: copper; Fe: iron; K: potassium; Mg: magnesium; Mn: manganese; Mo: molybdenum; Na: sodium; P: phosphorus; and Zn: zinc

**Fig 1:**
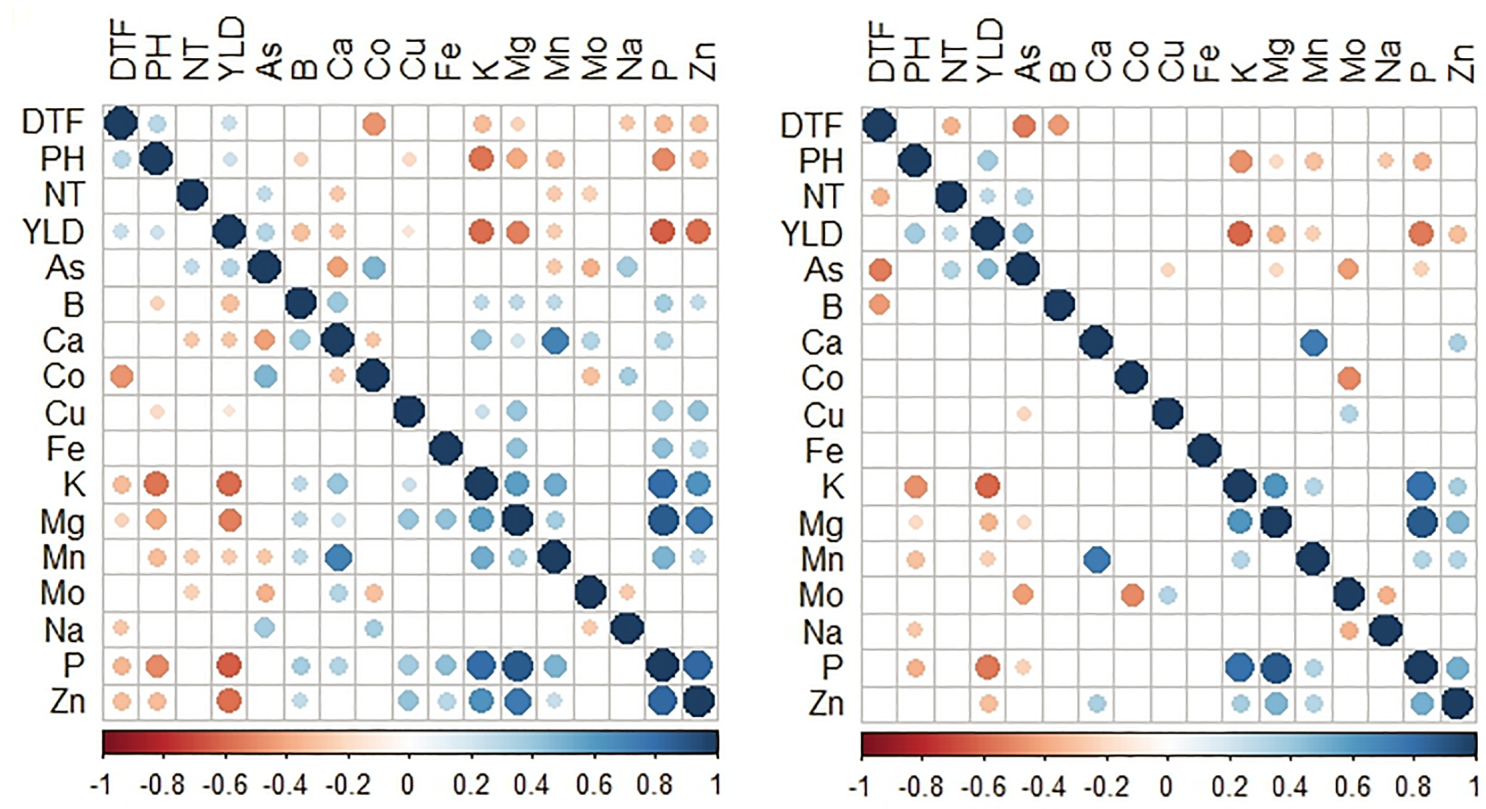
A heatmap depicting Pearson’s correlation coefficients between agronomic and micronutrient traits in P1 population for 2015WS and DS. Circle size indicates significant correlations using a two-paired *t*-test (*n* = 130 DH lines).

**Fig 2:**
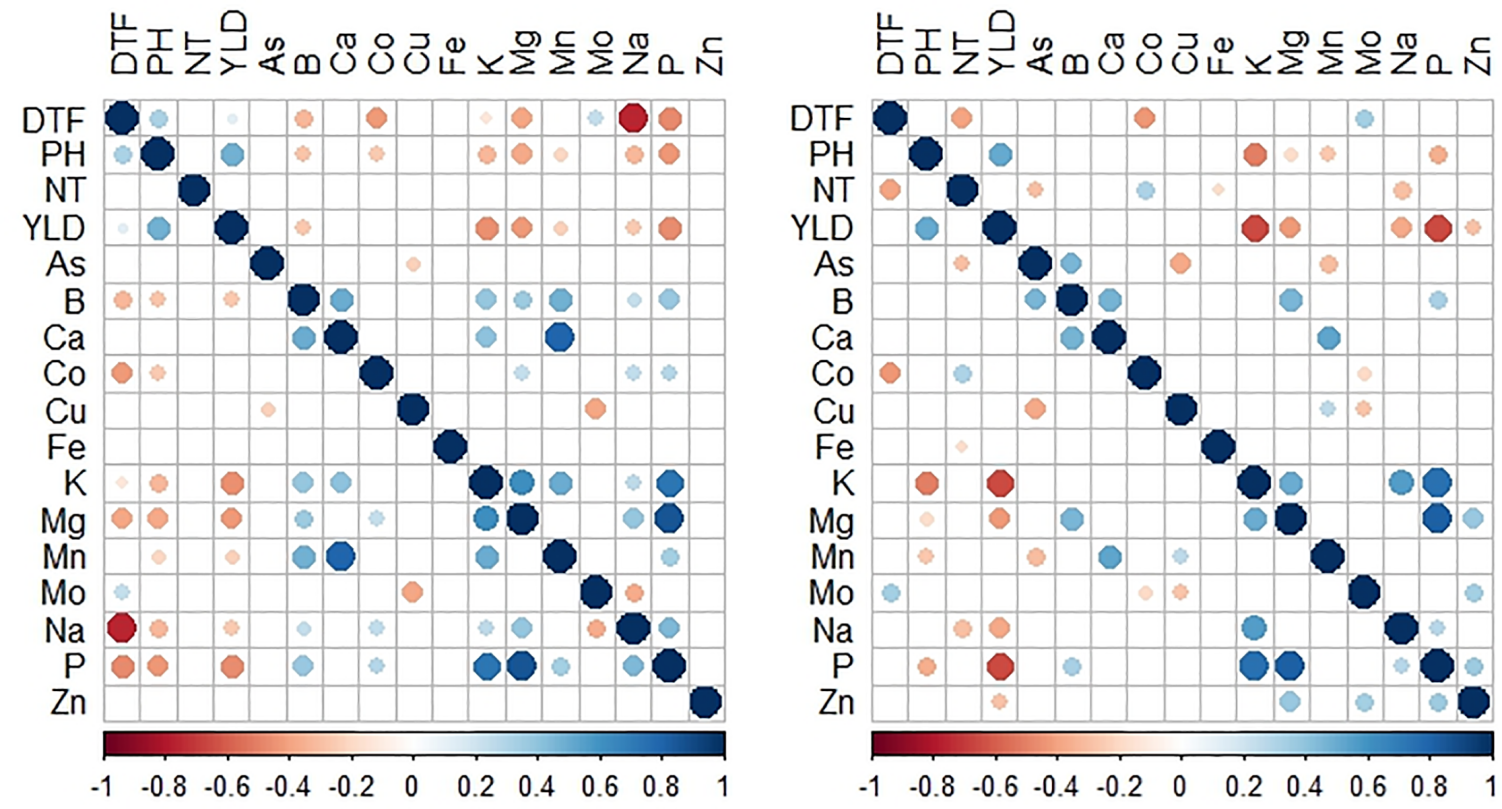
A heatmap depicting Pearson’s correlation coefficients between agronomic and micronutrient traits in P2 population for 2015WS and DS. Circle size indicates significant correlations using a two-paired *t*-test (*n* = 97 DH lines).

Pearson correlation results indicated that the number of significant correlations between traits was higher in the DS compared to the WS (Figs 1 and 2). Similarly, the number of positive correlations was higher in P1. Notable significant positive correlations were observed among phenotypic traits; including PH and YLD, and NT and YLD, among biofortification traits between Zn and Fe; while highly significant negative correlation was observed between YLD and Zn, and YLD and Fe. Furthermore, positive and negative significant correlations were also observed in other mineral elements. For example, Fe and Zn were positively correlated with other mineral elements such as Mg, Co, Cu, K, Ca, P, B, Mo, and Mn except Na and As, while negative correlations were found among NT, PH, DTF, and YLD.

### SNP-based molecular map construction

Of the 6,404 SNP markers used to genotype the parents and progenies of each population, 38.2% were polymorphic between parents of P1; while 33.2% were polymorphic between parents of P2. Removal of redundant markers resulted in 469 SNP markers and 398 SNP markers remaining for linkage map construction for P1 and P2, respectively. The distribution of SNP markers varied across different chromosomes (S1 Table). The highest and the lowest numbers of SNPs were on chromosomes 2 (65) and 12 (10), respectively in P1, while the highest and the lowest numbers of SNPs were on chromosomes 3 (86) and 8 (10), respectively in P2. The total lengths of the linkage maps of P1 and P2 were 1396.5 and 1619.2 cM, respectively. The average marker intervals were 2.98cM and 4.07 cM in P1 and P2, respectively.

### QTL analysis

**Fig 3:**
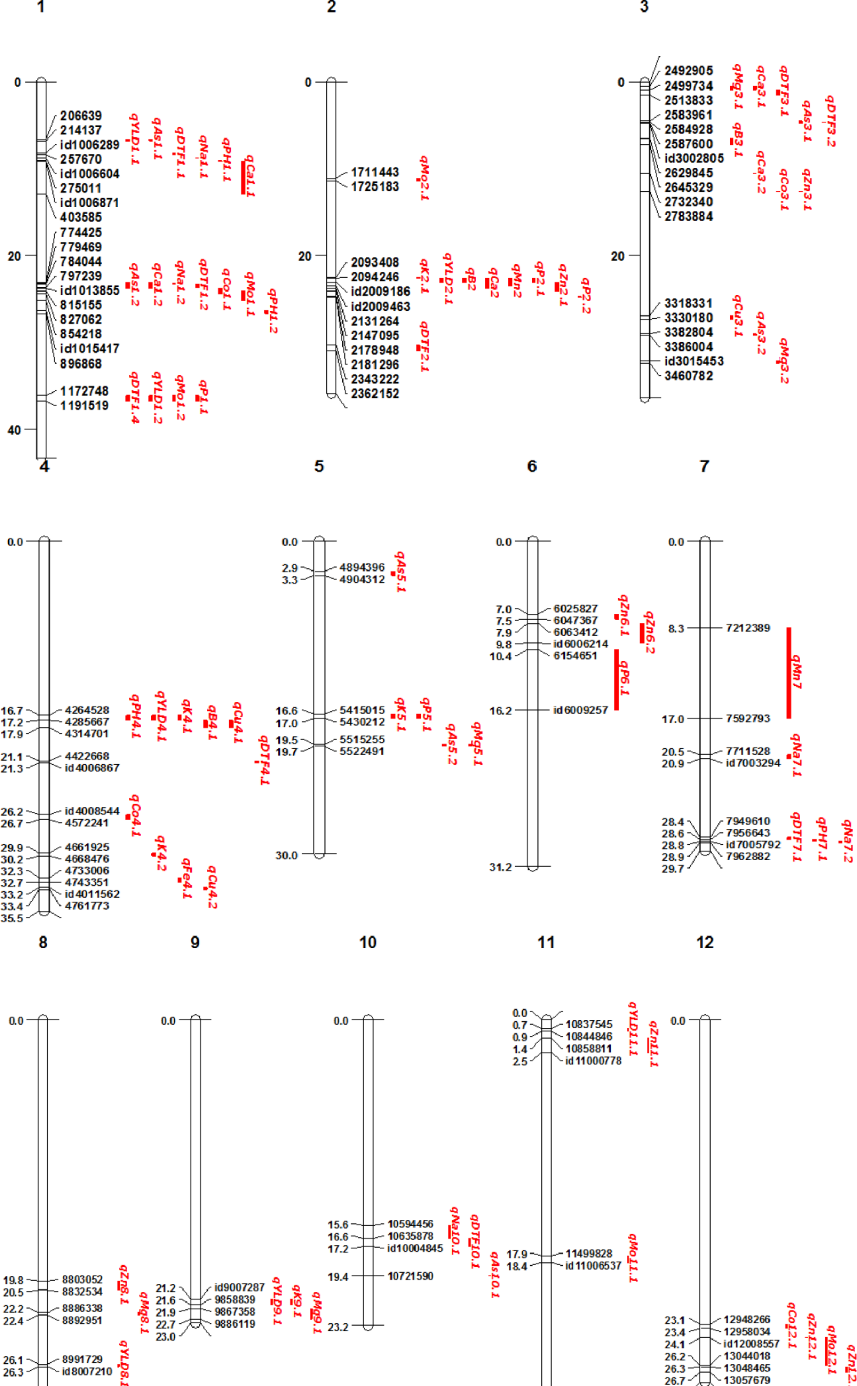
Physical locations of QTLs and candidate genes associated with agronomic and grain micronutrients traits in P1 and P2 during two seasons. Candidate genes in red are either within or <0.1 Mb of QTLs.

QTL analysis identified 79 QTLs for 16 traits during 2015DS and WS in both P1 and P2 (Table 2 and Fig 3). Most of the QTLs contributed more than 10% of the total phenotypic variance explained (PVE) and also had a high additive effect on the traits. The details of the QTLs identified are provided in Table 2.

**Table 2:** Details of QTLs for the different traits in P1 and P2 during 2015DS and WS.

Trait	QTL	Ch	Position (cM)	LM	RM	LOD	PVE (%)	Add	Season	Allele	Pop
**DTF**	*qDTF*_*1*.*1*_	1	23	id1006289	257670	5.8	11.1	2.2	DS	PSBRc82	1
	*qDTF*_*1*.*2*_	1	71	784044	797239	8.4	13.0	3.1	DS	PSBRc82	2
	*qDTF*_*1*.*2*_	1	71	784044	797239	4.9	15.0	3.2	WS	PSBRc82	2
	*qDTF*_*1*.*3*_	1	121	1172748	1191519	12.8	20.3	-4.5	WS	Joryeongbyeo	1
	*qDTF*_*2*.*1*_	2	127	2343222	2362152	7.4	10.8	2.7	WS	PSBRc82	1
	*qDTF*_*3*.*1*_	3	6	id3000480	2513833	10.2	16.7	-3.3	DS	IR69428	2
	*qDTF*_*3*.*2*_	3	80	2584928	2587600	3.3	9.4	-2.3	WS	IR69428	2
	*qDTF*_*4*.*1*_	4	54	4422668	id4006867	9.6	14.2	-2.5	WS	Joryeongbyeo	1
	*qDTF*_*7*.*1*_	7	139	7949610	7956643	9.3	14.3	-3.0	DS	IR69428	2
	*qDTF*_*7*.*1*_	7	137	7949610	7956643	4.3	12.3	-2.7	WS	IR69428	2
	*qDTF*_*10*.*1*_	10	107	10635878	id10004845	12.0	20.3	-3.8	DS	IR69428	2
	*qDTF*_*10*.*1*_	10	107	10635878	id10004845	6.9	21.5	-3.7	WS	IR69428	2
**PH**	*qPH*_*1*.*1*_	1	30	275011	id1006871	5.9	12.4	4.7	DS	PSBRc82	1
	*qPH*_*1*.*2*_	1	87	id1015417	896868	6.7	15.3	5.7	WS	PSBRc82	1
	*qPH*_*4*.*1*_	4	0	4264528	4285667	6.1	12.6	4.2	DS	PSBRc82	1
	*qPH*_*4*.*1*_	4	1	4264528	4285667	7.9	18.7	6.3	WS	PSBRc82	1
	*qPH*_*7*.*1*_	7	146	7956643	id7005792	3.4	15.3	-3.8	DS	IR69428	2
**YLD**	*qYLD*_*1*.*1*_	1	14	206639	214137	3.5	5.2	-244.3	WS	Joryeongbyeo	1
	*qYLD*_*1*.*2*_	1	121	1172748	1191519	10.2	17.0	485.8	WS	PSBRc82	1
	*qYLD*_*2*.*1*_	2	6	2094246	id2009186	5.4	17.4	527.6	DS	PSBRc82	1
	*qYLD*_*4*.*1*_	4	0	4264528	4285667	15.2	27.5	439.6	WS	PSBRc82	1
	*qYLD*_*8*.*1*_	8	43	8991729	id8007210	4.7	11.7	415.0	WS	PSBRc82	2
	*qYLD*_*9*.*1*_	9	165	id9007287	9858839	7.1	18.9	639.7	WS	PSBRc82	2
	*qYLD*_*11*.*1*_	11	0	10837545	10844846	4.8	11.9	544.3	WS	PSBRc82	2
**As**	*qAs*_*1*.*1*_	1	11	206639	214137	11.2	27.0	-0.032	DS	Joryeongbyeo	1
	*qAs*_*1*.*2*_	1	63	774425	id1013855	5.8	12.5	-0.015	WS	Joryeongbyeo	1
	*qAs*_*3*.*1*_	3	29	2583961	id3002805	5.2	10.7	0.015	DS	PSBRc82	1
	*qAs*_*3*.*2*_	3	245	3382804	3386004	4.0	14.4	-0.018	WS	IR69428	2
	*qAs*_*5*.*1*_	5	74	4894396	4904312	4.4	22.8	-0.028	DS	IR69428	2
	*qAs*_*5*.*2*_	5	87	5515255	5522491	7.1	15.5	0.016	WS	PSB Rc82	1
	*qAs*_*10*.*1*_	10	124	10721590	10722207	7.0	25.8	-0.024	WS	IR69428	2
**B**	*qB*_*2*.*1*_	2	5	2094246	id2009186	9.7	17.1	-0.357	WS	Joryeongbyeo	1
	*qB*_*3*.*1*_	3	100	2629845	2645329	9.9	23.5	0.433	WS	PSBRc82	2
	*qB*_*4*.*1*_	4	17	4285667	4314701	13.3	24.0	0.414	WS	PSBRc82	1
**Ca**	*qCa*_*1*.*1*_	1	6	id1006871	403585	5.4	20.2	6.917	DS	PSBRc82	2
	*qCa*_*1*.*2*_	1	59	774425	id1013855	5.4	14.5	7.012	DS	PSBRc82	1
	*qCa*_*2*.*1*_	2	5	2094246	id2009186	40.4	37.0	-16.304	WS	Joryeongbyeo	1
	*qCa*_*2*.*1*_	2	21	id2009463	2131264	10.1	31.0	-10.006	DS	Joryeongbyeo	1
	*qCa*_*3*.*1*_	3	2	2493147	2499734	3.9	13.1	5.501	DS	PSBRc82	2
	*qCa*_*3*.*2*_	3	128	2732340	2733626	4.5	15.4	-6.006	DS	IR69428	2
**Co**	*qCo*_*1*.*1*_	1	67	id1013855	827062	15.8	29.2	-0.008	WS	Joryeongbyeo	1
	*qCo*_*3*.*1*_	3	149	2783884	2785595	7.0	21.5	0.006	WS	PSBRc82	2
	*qCo*_*4*.*1*_	4	114	id4008544	4572241	10.8	29.2	0.003	DS	PSBRc82	1
	*qCo*_*12*.*1*_	12	59	12948266	12958034	4.5	23.2	0.003	DS	PSBRc82	2
**Cu**	*qCu*_*3*.*1*_	3	213	3318331	3330180	6.1	21.2	-0.379	DS	IR69428	2
	*qCu*_*4*.*1*_	4	17	4285667	4314701	31.8	30.4	-0.655	WS	Joryeongbyeo	1
	*qCu*_*4*.*2*_	4	98	id4011562	4761773	3.8	12.1	-0.287	DS	IR69428	2
**Fe**	*qFe*_*4*.*1*_	4	178	4733006	4743351	3.2	9.4	0.4	WS	PSBRc82	1
**K**	*qK*_*2*.*1*_	2	4	2093408	2094246	5.5	21.7	-131.943	DS	Joryeongbyeo	1
	*qK*_*4*.*1*_	4	7	4264528	4285667	9.1	15.5	-116.528	WS	Joryeongbyeo	1
	*qK*_*4*.*2*_	4	25	4661925	4668476	5.5	24.1	-173.529	DS	IR69428	2
	*qK*_*5*.*1*_	5	71	5415015	5430212	9.1	15.4	-119.413	WS	Joryeongbyeo	1
	*qK*_*9*.*1*_	9	164	id9007287	9858839	3.7	17.0	-139.711	WS	IR69428	2
**Mg**	*qMg*_*3*.*1*_	3	0	2492905	2499734	8.0	15.3	-35.529	WS	Joryeongbyeo	1
	*qMg*_*3*.*2*_	3	274	id3015453	3460782	5.4	24.3	-45.912	WS	IR69428	2
	*qMg*_*5*.*1*_	5	87	5515255	5522491	7.7	14.8	-39.389	WS	Joryeongbyeo	1
	*qMg*_*8*.*1*_	8	73	8886338	8892951	8.1	13.4	-71.013	DS	Joryeongbyeo	1
	*qMg*_*9*.*1*_	9	118	9867358	9886119	8.1	15.8	39.584	WS	PSBRc82	1
**Mn**	*qMn*_*2*.*1*_	2	5	2094246	id2009186	59.5	43.0	-6.174	WS	Joryeongbyeo	1
	*qMn*_*2*.*1*_	2	19	2110566	id2009463	8.5	18.6	-2.378	DS	Joryeongbyeo	1
	*qMn*_*7*.*1*_	7	25	7212389	7592793	10.3	23.6	-4.663	DS	Joryeongbyeo	1
**Mo**	*qMo*_*1*.*1*_	1	80	id1014853	854218	20.1	51.2	0.599	DS	PSBRc82	1
	*qMo*_*1*.*1*_	1	80	id1014853	854218	29.2	42.8	0.417	WS	PSBRc82	1
	*qMo*_*1*.*2*_	1	88	815155	id1014853	24.3	31.5	0.329	WS	PSBRc82	2
	*qMo*_*1*.*3*_	1	121	1172748	1191519	5.4	9.4	-0.374	DS	Joryeongbyeo	1
	*qMo*_*1*.*3*_	1	121	1172748	1191519	14.3	15.5	-0.339	WS	Joryeongbyeo	1
	*qMo*_*2*.*1*_	2	64	1711443	1725183	11.3	10.4	-0.196	WS	IR69428	2
	*qMo*_*11*.*1*_	11	49	11499828	id11006537	5.0	13.8	-0.265	DS	IR69428	2
	*qMo*_*12*.*1*_	12	14	12985052	13030749	7.2	6.8	-0.177	WS	Joryeongbyeo	1
	*qMo*_*12*.*1*_	12	90	13012866	13030749	4.4	12.5	0.193	DS	PSBRc82	2
	*qMo*_*12*.*1*_	12	95	13030749	13044018	9.3	8.0	0.183	WS	PSBRc82	2
**Na**	*qNa*_*1*.*1*_	1	26	id1006604	267954	10.7	30.7	-5.329	DS	Joryeongbyeo	1
	*qNa*_*1*.*2*_	1	70	779469	784044	7.6	23.7	-3.063	DS	IR69428	2
	*qNa*_*7*.*1*_	7	37	7711528	id7003294	4.8	12.4	-3.511	DS	Joryeongbyeo	1
	*qNa*_*7*.*2*_	7	149	id7005792	7962882	3.6	9.9	1.949	DS	PSBRc82	2
	*qNa*_*10*.*1*_	10	105	10594456	10635878	8.4	26.7	3.317	DS	PSBRc82	2
**P**	*qP*_*1*.*1*_	1	121	1172748	1191519	4.2	10.1	-140.294	WS	Joryeongbyeo	1
	*qP*_*2*.*1*_	2	6	2094246	id2009186	6.0	21.4	-177.609	DS	Joryeongbyeo	1
	*qP*_*2*.*2*_	2	39	2178948	2181296	5.6	13.5	-152.674	WS	Joryeongbyeo	1
	*qP*_*5*.*1*_	5	71	5415015	5430212	8.3	21.2	-140.149	WS	Joryeongbyeo	1
	*qP*_*6*.*1*_	6	84	6154651	id6009257	3.5	11.9	-174.877	DS	Joryeongbyeo	1
**Zn**	*qZn*_*2*.*1*_	2	15	2110566	id2009463	5.7	17.3	-1.0	WS	Joryeongbyeo	1
	*qZn*_*2*.*1*_	2	29	2140834	2147095	7.0	10.3	-1.4	DS	Joryeongbyeo	1
	*qZn*_*3*.*1*_	3	149	2783884	2785595	4.6	20.3	1.0	DS	PSBRc82	2
	*qZn*_*6*.*1*_	6	73	6025827	6047367	5.3	15.3	-1.5	WS	Joryeongbyeo	1
	*qZn*_*6*.*2*_	6	80	6063412	id6006214	10.3	16.1	-2.1	DS	Joryeongbyeo	1
	*qZn*_*8*.*1*_	8	64	8803052	8832534	9.2	14.1	-1.4	DS	Joryeongbyeo	1
	*qZn*_*11*.*1*_	11	10	10858811	id11000778	5.1	22.8	-1.6	WS	IR69428	2
	*qZn*_*12*.*1*_	12	8	id12008557	12985052	5.2	7.5	1.1	DS	PSB Rc82	1
	*qZn*_*12*.*2*_	12	23	13048465	13057679	4.3	12.2	-0.9	WS	Joryeongbyeo	1

### QTLs identified for agronomic traits

**DTF:** In all, nine QTLs for DTF located on chromosomes 1, 2, 3, 4, 7, and 10 were identified in P1 and P2. The PVE by these QTLs ranged from 9.4% to 21.5%. Three QTLs (*qDTF*_*1*.*2*_, *qDTF*_*7*.*1*._, and *qDTF*_*10*.*1*_*)* with a large and consistent effects located on chromosomes 1, 7, and 10 were identified in P2, while six QTLs (*qDTF*_*1*.*1*_, *qDTF*_*1*.*3*_, *qDTF*_*2*.*1*_, *qDTF*_*3*.*1*_, *qDTF*_*3*.*2*_, and *qDTF*_*4*.*1*_) were specific to individual populations and seasons. The positive alleles for DTF were contributed by the high-yielding parent PSBRc82 for three QTLs (*qDTF*_*1*.*1*_, *qDTF*_*1*.*2*_, and *qDTF*_*2*.*1*_), while the paternal parents Joryeongbyeo and IR69428 contributed two QTLs (*qDTF*_*1*.*3*_ and *qDTF*_*4*.*1*_) and four QTLs (*qDTF*_*3*.*1*_, *qDTF*_*3*.*2*_, *qDTF*_*7*.*1*_, and *qDTF*_*10*.*1*_), respectively.

**PH:** There were four QTLs identified for PH, three of which were detected in P1 and only one QTL was identified in P2. The QTLs *qPH*_*1*.*1*_ and *qPH*_*1*.*2*_ were identified on chromosome 1, and one QTL each (*qPH*_*4*.*1*_ and *qPH*_*7*.*1*_) were identified on chromosomes 4 and 7, respectively. All the QTLs for PH have PVE that ranged from 12.4% to 18.7% and contributed an additive effects equivalent from 3.8 to 6.3 cm. All QTLs were derived from the maternal parent except *qPH*_*7*.*1*_. QTL *qPH*_*4*.*1*_ was consistently identified during both seasons in P1.

**YLD:** Seven QTLs on chromosomes 1, 2, 4, 8, 9, and 11 were identified for grain yield in P1 and P2 during two seasons. These QTLs have PVE that ranged from 5.2% to 27.5% and were derived from high-yielding parent except *qYLD*_*1*.*1*_. Further, all QTLs for yield were identified in the WS except *qYLD*_*2*.*1*_.

#### QTLs for grain element concentration

**As:** Seven QTLs were detected on chromosomes 1, 3, 5, and 10. These QTLs explained from 10.7 to 27% of the phenotypic variance and the additive effects varied from 0.01 to 0.03 ppm. Two of the seven QTLs were derived from the high-yielding parent, PSBRc82

**B:** Three QTLs were located on chromosomes 2, 3, and 4. The QTLs have PVE that ranged from 17.1% to 24% and the additive effects ranged from 0.36 to 0.43 ppm and were detected only during the WS. Two of them (*qB*_*3*.*1*_ and *qB*_*4*.*1*_) were derived from the maternal parent, PSBRc82, while *qB*_*2*.*1*_ was derived from the paternal parent, Joryeongbyeo.

**Ca:** Five QTLs were identified on chromosomes 1, 2, and 3 with PVE that ranged from 13.1% to 37.0% and additive effects that ranged from 5.5 to 16.3 ppm. All QTLs for Ca were detected in the DS, while *qCa*_*2*.*1*_ was consistent across both DS and WS and was derived from the paternal parent in P1.

**Co:** Four QTLs were detected on chromosomes 1, 3, 4, and 12. The PVE of the QTLs ranged from 21.5% to 29.2% and the additive effects varied from 0.003 to 0.008 ppm. All QTLs except *qCo*_*1*.*1*_ were derived from the high-yielding parent, PSBRc82.

**Cu:** Three QTLs on chromosomes 3 and 4 derived from the paternal parents, IR69428 and Joryeongbyeo, were identified. The PVE of these QTLs ranged from 12.1% to 30.4% and have additive effects that ranged from 0.29 to 0.66 ppm.

**Fe:** One QTL, *qFe*_*4*.*1*_, with a PVE of 9.4% and an additive effect of 0.4 ppm, was detected on chromosome 4. It was derived from the high-yielding parent, PSBRc82, identified during the WS.

**K:** Five QTLs on chromosomes 2, 4, 5, and 9 were identified. All of them were derived from the paternal parents, IR69428 and Joryeongbyeo. The PVE ranged from 15.4% to 24.1% and the additive effects ranged from 116.5 to 173.5 ppm.

**Mg:** Five QTLs were identified on chromosomes 3, 5, 8, and 9. The PVE ranged from 13.4% to 24.3% and the additive effects ranged from 35.5 to 71.0 ppm. Four QTLs were derived from the paternal parents, IR69428 (*qMg*_*3*.*2*_) and Joryeongbyeo (*qMg*_*3*.*1*_, *qMg*_*5*.*1*_, *qMg*_*8*.*1*_) while only one QTL was derived from the maternal parent, PSBRc82 (*qMg*_*9*.*1*_).

**Mn:** Two QTLs were identified on chromosomes 2 and 7. The PVE ranged from 18.6% to 43% and the additive effects ranged from 2.4 to 6.2 ppm. The QTL *qMn*_*2*.*1*_ was identified in P1 across both seasons.

**Mo:** Six QTLs were detected on chromosomes 1, 2, 11, and 12. The PVE ranged from 6.8% to 51.2% and the additive effects ranged from 0.18 to 0.6 ppm. QTL *qMo*_*12*.*1*_ was identified in both populations and was consistent across seasons in P2. Three of the six QTLs were contributed by the paternal parents, IR69428 and Joryeongbyeo.

**Na:** Five QTLs were detected on chromosomes 1, 7, and 10, all of which were identified only in the DS. The PVE ranged from 9.9% to 30.7% and the additive effects ranged from 1.95 to 5.33 ppm.

**P:** Five QTLs were detected for P on chromosomes 1, 2, 5, and 6, and were derived from Joryeongbyeo. The PVE ranged from 10.1% to 21.4% and the additive effects ranged from 140.2 to 177.6 ppm.

**Zn:** Eight QTLs were identified on chromosomes 2, 3, 6, 8, 11, and 12. The PVE ranged 7.5% to 22.8% and the additive effects ranged from 0.9 to 2.1 ppm. The QTL *qZn*_*2*.*1*_ was consistently identified in both seasons and was derived from Joryeongbyeo. Six QTLs (*qZn*_*2*.*1*_, *qZn*_*6*.*1*_, *qZn*_*6*.*2*_, *qZn*_*8*.*1*_, *qZn*_*11*.*1*_, and *qZn*_*12*.*2*_) were derived from the paternal parents, IR69428 and Joryeongbyeo, whereas two QTLs (*qZn*_*3*.*1*_ and *qZn*_*12*.*1*_) were derived from the maternal parent, PSBRc82.

QTLs that are consistent across seasons and those with large effects (PVE>25%) could be targeted for marker-assisted selection. We have identified 79 QTLs for agronomic and biofortification traits. Of these, six QTLs were consistent across seasons in P1 (*qPH*_*4*.*1*_, *qCa*_*2*.*1*_, *qMn*_*2*.*1*_, *qMo*_*1*.*1*_, *qMo*_*1*.*3*_, and *qZn*_*2*.*1*_), three were detected across seasons in P2 (*qDTF*_*1*.*2*_, *qDTF*_*7*.*1*_, *qDTF*_*10*.*1*_). The QTL *qMo*_*12*.*1*_ was identified in both populations and was consistent across seasons in P2. Further, 12 QTLs have large effects: *qYLD*_*4*.*1*_, *qAs*_*1*.*1*_, *qAs*_*10*.*1*_, *qCa*_*2*.*1*_, *qCo*_*1*.*1*_, *qCo*_*4*.*1*_, *qCu*_*4*.*1*_, *qMn*_*2*.*1*_, *qMo*_*1*.*1*_, *qMo*_*1*.*2*_, *qNa*_*1*.*1*_, and *qNa*_*10*.*1*_. It is also noteworthy that *qCa*_*2*.*1*_, *qMn*_*2*.*1*_, and *qMo*_*1*.*1*_ are large-effect QTLs detected in P1 and consistent across seasons.

#### Co-location of QTLs for agronomic and biofortification traits

In all, fourteen QTL clusters each consisting of two to six QTLs were identified (S2 Table and Fig 3). Four major QTL clusters consisting of at least four QTLs were observed on chromosomes 1, 2, and 4. The first of which, located between 23.08 Mb to 23.7 Mb on chromosome 1 and it included four QTLs, *qDTF*_*1*.*2*_, *qAs*_*1*.*2*_, *qCa*_*1*.*2*_, and *qNa*_*1*.*2*_. The QTL cluster located ~36.1 to 36.8 Mb on chromosome 1 included four QTLs, *qDTF*_*1*.*4*_, *qYLD*_*1*.*2*_, *qMo*_*1*.*3*_, and *qP*_*1*.*1*_. The QTL cluster located from 22.5 to 24.1 Mb on chromosome 2 includes six QTLs, *qYLD*_*2*.*1*_, *qB*_*2*.*1*_, *qCa*_*2*.*1*_, *qK*_*2*.*1*_, *qMn*_*2*.*1*_, and *qZn*_*2*.*1*_. Meanwhile, the QTL cluster located ~16.7 to 18.0 Mb on chromosome 4 includes five QTLs such as *qPH*_*4*.*1*_, *qYLD*_*4*.*1*_, *qB*_*4*.*1*_, *qCu*_*4*.*1*_, and *qK*_*4*.*1*_. Some of the other major QTL co-locations such as *qYLD*_*1*.*1*_ and *qAs*_*1*.*1;*_ *qCa*_*1*.*1*_ and *qMo*_*1*_; *qCo*_*1*.*1*_ and *qMo*_*1*.*2*_ on chromosome 1. Similarly *qDTF*_*3*.*1*_ and *qMg*_*3*.*1;*_ *qDTF*_*3*.*2*_, *qAs*_*3*.*1*_ and *qCa*_*3*.*1;*_ *qCo*_*3*.*1*_ and *qZn*_*3*.*1*_ on chromosome 3; *qK*_*5*.*1*_ and *qP*_*5*.*1*_; *qAs*_*5*.*2*_ and *qMg*_*5*.*1*_ on chromosome 5; *qDTF*_*7*.*1*_ and *qPH*_*7*.*1*_ on chromosome 7; *qYLD*_*9*.*1*_ and *qK*_*9*.*1*_, and *qMg*_*9*.*1*_ on chromosome 9 were found to be co-located.

A number of correlated traits share common QTLs detected in the same population and season. The negatively correlated YLD and P share common QTLs on chromosomes 1 and 2 detected in P1 during the WS. The positively correlated K and P have common QTLs on chromosome 2 detected in P1 during the DS and on chromosome 5 detected in the same population during the WS. Both K and P are negatively correlated with YLD; these three traits share a common QTL on chromosome 2 detected in P1 during the DS. The positively correlated Ca, Mn, and Zn share a common QTL on chromosome 2 identified in P1 during the WS. The positively correlated PH and YLD were both negatively correlated to K, these three sharing a common QTL on chromosome 4 identified in P1 during the WS. The negatively correlated As and Mg share a common QTL on chromosome 5 detected in P1 during the WS. The negatively correlated DTF and PH share a common QTL on chromosome 7 uncovered in P2 during DS. The negatively correlated YLD and K share a common QTL on chromosome 9 detected in P2 during WS.

### Epistasis for agronomic and biofortification traits

Eight di-genic interactions were detected in P2 for Cu, Mg, Na, and Zn while no interactions were observed in P1 (Table 3). Three epistatic interactions between loci on chromosomes 3, 8, and 10 for Na explained ~28.6% PVE each. One epistatic interaction was identified for Mg between loci on chromosomes 1 and 7 that contributed 34.1% of the PVE. Two epistatic interactions for Cu, detected on chromosome 5 and between chromosomes 8 and 9, accounted for 43.2 and 28.1% of the PVE, respectively. For Zn, 2 epistatic interactions were identified that contributed 16.9 and 32.6% of the PVE, one between loci on chromosomes 5 and 9 and the other between loci on chromosomes 3 and 5.

**Table 3:** Epistasis analysis for different traits in P2.

Traits	Chr	QTL (Ai)			QTL (Aj)		LOD	PVE(%)	AiAj
		Interval		Chr	Interval				
		Pos 1	Pos 2		Pos 1	Pos 2			
Cu	8	8926819	8940497	9	id9005523	9739786	7.21	43.22	0.52
Cu	5	5017498	id5003312	5	id5007247	5477886	9.65	28.17	-3.69
Mg	1	815155	id1014853	7	7750124	id7002196	6.05	34.14	-59.36
Na	3	2572256	2580102	3	2584928	2587600	50.91	28.65	-36.1
Na	8	8940497	8951753	8	id8006885	8966923	52.83	28.56	-35.1
Na	10	10032781	10060149	10	10154319	10259438	48.6	28.53	-35.34
Zn	5	4994496	5009150	9	id9007287	9858839	6.35	16.89	1.54
Zn	3	id3001869	2572256	5	5017498	id5003312	6.27	32.61	-1.12

**AiAj:** additive x additive interaction; **PVE:** Phenotypic Variance Explained

### Candidate genes located near associated SNPs

Candidate genes located within and near (<3.0 Mb) 43 QTLs of agronomic and biofortification traits were identified (S3 Table). For DTF, six main candidate genes *OsCCT01*, *HESO1* homolog, *OsLAX1*, *OsHAP3E*, *OscpSRP43* and *Ef2* were identified. Meanwhile, *OsSPL16* was main candidate gene, identified for grain yield.

For micronutrient traits, the primary candidate genes are those involved in metal and cation transport and those with transferase activity such as *OsALA2*, *OsAHA8*, and *OsECA1* for Ca; *OsMTP6*, *SOD*, and *OsNRAMP7* for Co; *OsSTA107* and *OsCOPT4* for Cu; *OsHAK1*, *OsGLR3*.*1*, *OsHKT4*, *OsHKT7*, *OsHKT15*, *OsHAK11*, and *OsHAK18* for K; *OsMRS2-5* and *OsMRS2-7* for Mg; *OsNRAMP1* and *OsNRAMP5* for Mn; *OsEFCAX1* and *OsHKT8* for Na; *OsPAP9b*, *OsABC1-2*, and *OsPPDKB* for P. One candidate gene *OsACR2*.*1* is involved in As metabolism. Other candidate genes are involved in response and sensitivity to micronutrients such as *OsDJ-1A*, *OsDJ-1B*, and several *OsSultr* genes for As; *OsCDT1* and *OsCBSCLC4* for Cu; and *OsOCP* for Fe. Others are involved in calcium-mediated signaling and detection such as *OsCam1-3*, *OsCam3*, and *OsCBL5*.

For Zn, the primary candidate genes were *OsMHX1*, *OsYSL2*, *OsMTP6*, *OsMTP12*, *OsTOM1*, *OsTOM2*, *OsZIFL7*, and *OsZIFL8*, and their functions are mainly for Zn ion transport as well as *OsNAS1* and *OsNAS2* that are involved in mediating the uptake of Zn from the soil. Other candidate genes are involved in response and sensitivity to the Zn ion such as *OsPDIL1-3*, *OsYSL15*, and *OsFER1*.

### Breeding lines with high yield and grain Zn

Eight breeding lines (Four lines per population) were identified as having high yield and high grain Zn based on their field performance (Table 4). All identified lines were comparable with IR64 in terms of yield for both seasons. Meanwhile, six of the eight lines consistently exhibited comparable Zn with check IR69428 during both DS and WS while two lines were only comparable with the check IR69428 during WS. Upon closer examination, each of the eight lines with high grain Zn and yield harbored one to three Zn QTLs, which account for from 14 to 50% of the Zn QTLs identified for each respective population. Of the seven Zn QTLs uncovered in P1, three were identified in IR91143-AC 89–1 while the other three lines either have one or two Zn QTLs. Meanwhile, of the two Zn QTLs uncovered in P2, only one was identified in the four lines.

**Table 4:** Mean performances of breeding lines with high yield and grain Zn from P1 and P2 populations in two seasons.

Designation	DS				WS				Pop	QTLs
	DTF	PH (cm)	YLD (kg/ha)	Zn (ppm)	DTF	PH (cm)	YLD (kg/ha)	Zn (ppm)		
IR 91143-AC 24–1	78	81	4782	17.6*	82	83	2561	18.9	1	*qZn*_*12*..*1*,_ *qYLD*_*1*.*2*_
IR 91143-AC 89–1	78	91	6453	18.9	85	103	2577	20	1	*qZn*_*2*.*1*_, *qZn*_*8*.*1*_, *qZn*_*12*.*1*,_ *qYLD*_*1*.*1*_*qYLD*_*1*.*2*_, *qYLD*_*2*.*1*_, *qYLD*_*4*.*1*_
IR 91143-AC 122–3	80	68	5564	23	89	84	3088	19.6	1	*qZn*_*8*.*1*_, *qZn*_*12*.*1*_, *qYLD*_*1*.*2*_, *qYLD*_*2*.*1*_, *qYLD*_*4*.*1*_
IR 91143-AC 290–1	79	84	5715	17.3*	89	85	2605	17.3	1	*qZn*_*8*.*1*_, *qZn*_*12*.*1*_, *qYLD*_*1*.*1*_, *qYLD*_*1*.*2*_, *qYLD*_*2*.*1*_, *qYLD*_*4*.*1*_
IR 85850-AC 120–1	77	84	4656	20.3	76	91	3334	17.2	2	*qZn*_*3*.*1*_, *qYLD*_*9*.*1*_
IR 85850-AC 157–1	94	81	5992	20.5	93	87	3320	17.2	2	*qZn*_*3*.*1*_, *qYLD*_*8*.*1*_, *qYLD*_*9*.*1*_, *qYLD*_*11*.*1*_
IR 85850-AC 125–1	101	101	5060	18.8	101	104	2884	21.5	2	*qZn*_*3*.*1*_, *qYLD*_*9*.*1*_, *qYLD*_*11*.*1*_
IR 85850-AC 160–1	96	97	5421	20	94	94	2722	19.8	2	*qZn*_*3*.*1*_, *qYLD*_*8*.*1*_, *qYLD*_*9*.*1*_
IR 64	85	86	5176	15.9*	88	78	2499	13.5*		
IR69248	104	92	5574	22	101	91	2394	20.3		

*Significant pairwise comparison with Zn check (IR69428)

note: all 8 lines have comparable mean yields with yield check, IR64.

## Discussion

Biofortification is considered the most promising, food-based approach to address micronutrient malnutrition [26]. There have been significant efforts over the last decade to biofortify the major cereals, pulses, and tuber crops targeted to different parts of the world and a lot of progress has already been achieved in this endeavor [8]. Rice, the dominant cereal and a major staple in Asia, is the prime target for biofortification to tackle micronutrient malnutrition in South Asia and Southeast Asia [27]. In order to make progress in biofortification of rice, there is a requirement for enough variability in grain micronutrients in relevant germplasm collections, a better understanding of the genetic basis of the grain micronutrients, and their interactions with genetic backgrounds and environmental factors [12]. Recent advances in rice genomics have enabled breeders to dissect the molecular basis of complex grain micronutrient traits to identify QTLs/genes for use in MAB [28], [29], [30]. At IRRI, we developed and evaluated two DH populations and mapped QTLs for agronomic and grain micronutrients.

The analysis of variance indicated the significant genotype effects for most of the traits. The mean, range and CV also indicated wide variation for all the agronomic and grain micronutrients traits in both the populations and seasons. Also, most of the traits showed normal distributions indicating a complex genetic basis. It is a well-known fact that polygenically inherited complex traits, such as agronomic traits, yield, and grain micronutrient traits, show wide variation and are greatly affected by GxE [17]. Several earlier studies on analysis of multiple micronutrients in rice grains from germplasm collections and mapping populations evaluated in different environments provided similar results [3], [15], [28], [31], [32].

Moderate to high broad-sense heritability (H^2^>60%) was observed for most of the traits except for NT and Fe, suggesting that they are genetically controlled and amenable to genetic manipulation. A high H^2^ (>50%) and significant GxE for mineral elements, such as Zn, Cu, Mo, and Mg, observed across the populations and locations in most the recent studies on grain micronutrients [12], [17], [28]. The association results clearly show that there are positive correlations among different micronutrients, but they have a negative association with YLD; while Fe and Zn were strongly positively correlated irrespective of the seasons, locations, and populations. Several studies have reported the positive relationship between Fe and Zn [14], [33] as well as the negative relationship between YLD and Zn in rice [28], [34], [35]. However, there are some reports showing positive or no significant correlations between yield and Zn [36], [37]. A positive correlation between Fe and Zn could allow simultaneous improvement of both minerals [22]. However, the negative linkages between YLD and Zn must be eliminated for successful biofortification. Hence it is necessary to identify high-Zn parental lines with acceptable yield potential, by designing appropriate breeding strategies, selection schemes and evaluation procedures for the successful development and release of high-Zn rice varieties [38], [39].

The SNP marker analysis revealed high rates of polymorphism (>30%) between the parents, and this was expected because the paternal parents IR69428 (*japonica* derivative) and Joryeongbyeo *(japonica*) were genetically distant from PSBRc82 (a popular *indica* rice variety). Eventhough polymorphism was higher but the final set of markers used for linkage map construction were 469 SNP and 398 SNPs in the P1 and P2, this is mainly because we removed markers with ambiguous or missing allelic calls, redundant, unanchored and unlinked SNP markers. All these steps resulted in less number markers on some chromosomes leading to some gaps. However, over all the SNP set gave good genome coverage with a marker density of 2.98cM and 4.07 cM which was enough for the linkage map consutruction and QTL analysis in a DH population.

We detected 20 QTLs for agronomic traits and 59 QTLs for mineral elements, almost 40% of them were derived from the paternal parents. The most significant QTLs identified in our study were *qAs*_*1*.*1*_, *qAs*_*10*.*1*_, *qCa*_*2*.*1*_, *qCo*_*1*.*1*_, *qCo*_*4*.*1*_, *qCu*_*4*.*1*_, *qMn*_*2*.*1*_, *qMo*_*1*.*1*_, *qMo*_*1*.*2*_, *qNa*_*1*.*1*_, and *qNa*_*10*.*1*_ and each had a PVE of more than 25%. Allelic effects of the QTLs for Cu, K, Mn, and P were from either of the paternal parents (IR69428 or Joryeongbyeo), while Cu and P QTLs were specific to Joryeongbyeo. In general, most of the QTLs for YLD and PH were contributed by PSBRc82, while QTLs for mineral elements were contributed by the paternal parents. But only 10 QTLs were consistent across the seasons and most of them, such as *qCa*_*2*.*1*_, *qMn*_*2*.*1*_, *qMo*_*1*.*2*_, *Mo*_*1*.*3*_, *qMo*_*12*.*1*_, and *qZn*_*2*.*1*_, were detected only in P1. It is notable that *qMo*_*12*.*1*_ was the only QTL consistent across the populations and seasons, indicating the significant genetic background effect and significant GxE for all the other mineral elements. For YLD, all the QTLs except *qYLD*_*1*.*1*_ were contributed by PSBRc82, with a PVE of 5% and additive effect of 0.24 t ha^-1^. Conversely, 6 of the 8 QTLs for grain Zn were derived from paternal parents; all of them were season- and population-specific except *qZn*_*2*.*1*_. Recent studies on QTL mapping for mineral elements in rice and other crops using RIL, ILs, F_2_, DH, and MAGIC populations have identified multiple loci and clearly demonstrated the genetic complexity of the grain micronutrient traits [3], [14], [17]. Some of the loci identified in our study corresponded to the same chromosomal regions and traits as for other reports. However, thorough characterization and validation of the QTLs/genes are prerequisites before pursuing MAS. Since most of the QTLs are season and genetic background-specific, it will be necessary to pool multiple loci so that marker- assisted QTL pyramiding, marker-aided recurrent selection, or genomic selection can be appropriate strategies to develop rice varieties with improved micronutrient content.

Different mineral elements may share the same or similar pathways for their uptake, transport, and loading, consequently they may also share same genomic regions and QTL/genes. We identified several QTL clusters for multiple mineral elements and also for the agronomic traits. Present findings detected 14 co-localized QTLs responsible for different traits at specific genomic regions across chromosomes 1, 2, 3, 4, 5, 7, 9, and 12. Eleven of 14 co-localized QTLs were identified in both the WS and DS. Moreover, 4 of 11 were not only detected in both seasons, but also in both populations. Such trait co-locations have also been reported for Mg, Cu, Si, Se, Fe, K, Mn, and P [16], [28],[40]. Similarly, several clusters of QTLs were also found to be involved with grain minerals such as Zn, Fe, Mn, Zn/Fe, Mg, and Cu [41]. If the linked traits have positive correlations they can be simultaneously improved, however the negative linkages must be broken before their use in breeding.

Several epistatic QTLs were found for Cu, Mg, Na, and Zn with large effect QTLs contributing 16.9 to 43.2% of the PVE. Six epistatic QTLs identified for grain Zn accounted for 50.2% of the heritability of the trait [15]. Epistatic QTL for grain Zn with 20% of PVE was identified, which indicated strong genetic control involving many QTLs or genes [28]. Another epistatic QTL for Zn concentration was also observed explaining 60% of the total variance with an additive effect of 11.26 ppm [17]. A possible explanation for this observation may involve genetic changes wherein gene by gene interactions could modify the expression of phenotype and physiology [42]. Thus, they play a potential role in controlling grain mineral elements in polished rice.

The candidate gene analysis revealed that 16 known genes for agronomic traits and 52 candidate genes involved in metal homeostasis were found to co-locate with different QTLs identified in our study, emphasizing the importance of these loci for further molecular characterization or use in breeding. Some of the important gene families harboring the major QTLs are *OsNRAMP*, *OsZIP OsYSL*, *OsNAS*, *OsFER*, *OsZIFL*, and *OsOCP*. Their roles in metal uptake, transport, and loading are well characterized and their over-expression has shown a several-fold increase in mineral accumulation in different plant parts of rice [43–49]. Similarly, for agronomic traits, several important genes, such as *OsSPL14* and *OsSPL16*, were located near *qYLD*_*8*.*1*_. *OsSPL14*, which promotes panicle branching and higher grain productivity, might be useful for increasing grain yield in rice [50]. The expression of the *OsSPL16* gene has been shown to be involved in control of grain size, shape, and quality in rice [51].

The present study showed that several breeding DH lines with high grain Zn and grain yield were significantly high in both the populations as well as across the seasons. However, several previous studies have reported a significant negative relationship between grain Zn and grain yield in rice [28], [34], [35]. We identified eight DH lines with Zn more than 18 ppm with good agronomic traits. It is interesting note that among the DH lines IR91143-AC 122–3 showed yield and Zn content comparable to the parental line IR69428 but it had only two of the eight Zn QTLs. This may be due to the genetic background effect or transgressive variation due to epitasis and can be dissected further to understand the underlying mechanisms. These DH lines can be used as donors in breeding programs or can be directly tested in multi-location trials to further evaluate their performance.

## Conclusions

Considerable genetic variation for all traits was observed in the two DH populations. Seventy-nine QTLs were detected for agronomic and biofortification traits through inclusive composite interval mapping. The majority of the QTLs accounted for greater than 10% of the PVE. Grain Zn QTLs was found to cluster with QTLs for other biofortification traits, such as B, Ca, Mn, Mg, As, K, and Co. Six epistatic interactions accounted for a larger proportions of PVE ranging from 16.89 to 43.22%. Importantly, several candidate genes, such *as OsNRAMP*, *OsNAS*, *OsZIP*, *OsYSL*, *OsFER*, and *OsZIFL* were identified as necessary for grain Fe and Zn accumulation, together with *OsSPL14*, and *OsSPL16* for increasing grain yield.

## Supporting information

S1 Fig The frequency distribution of means of 130 P1 lines for yield/yield components and grain mineral nutrient contents. Red color: dry season (DS), green color: wet season (WS).
(JPG)

S2 Fig The frequency distribution of means of 97 DH lines for yield/yield components and grain mineral nutrient contents. Red color: dry season (DS), green color: wet season (WS).
(JPG)

S1 Table Details of mapping populations and linkage maps in two populations at each locus on 12 chromosomes. (XLSX)

S2 Table Colocalizated QTLs detected for all traits derived from P1 and P2 populations. (XLSX)

S3 Table Details of genes that might underlie QTLs for the different traits in P1 and P2 during 2015DS and WS. (XLSX)
