# Supplementary figures and images for: Identification of genomic regions associated with agronomic and biofortification traits in DH populations of rice
Source: PLoS One. 2018 Aug 10;13(8):e0201756. doi: 10.1371/journal.pone.0201756 (PMC6086416; doi:10.1371/journal.pone.0201756)

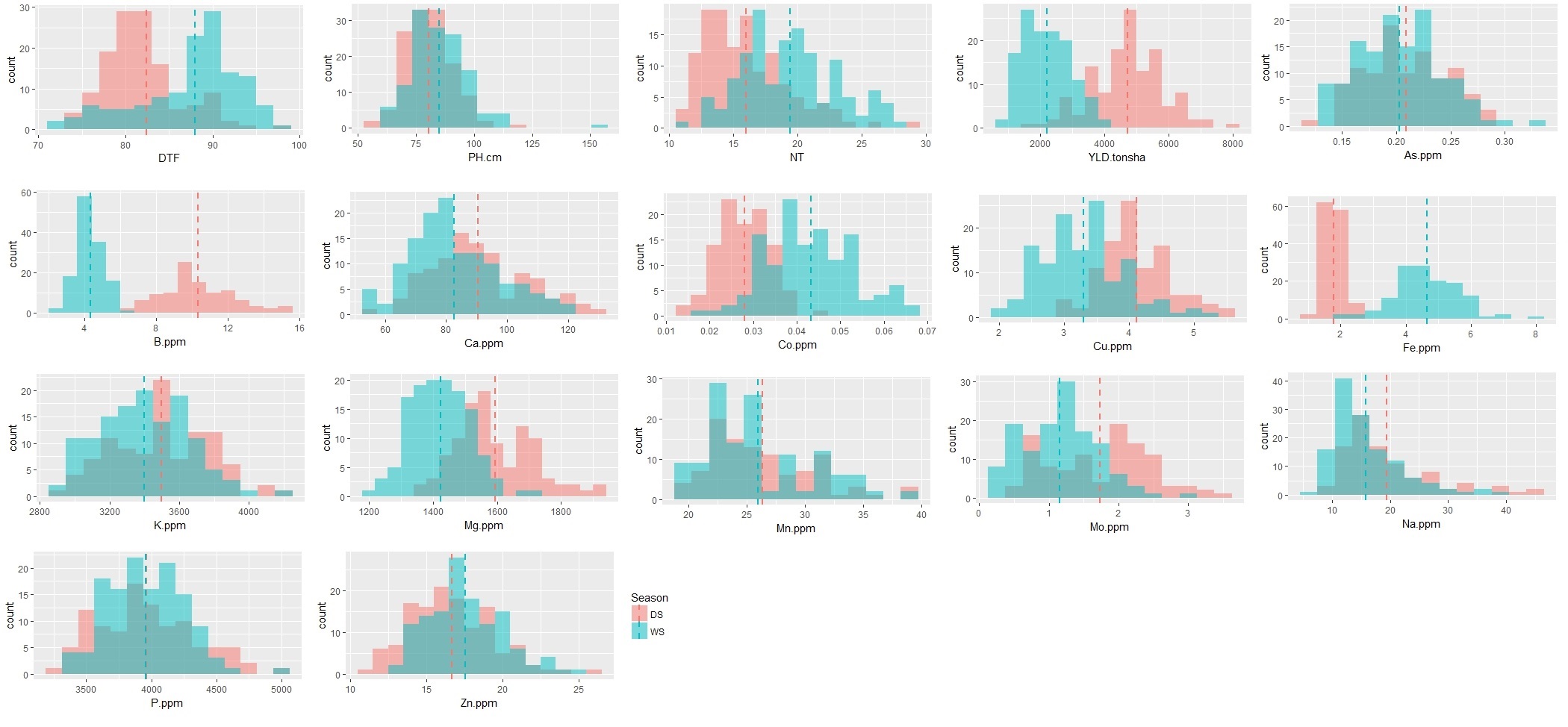

Supplement: S1 Fig — Red color: dry season (DS), green color: wet season (WS). (JPG) [file pone.0201756.s001.jpg]

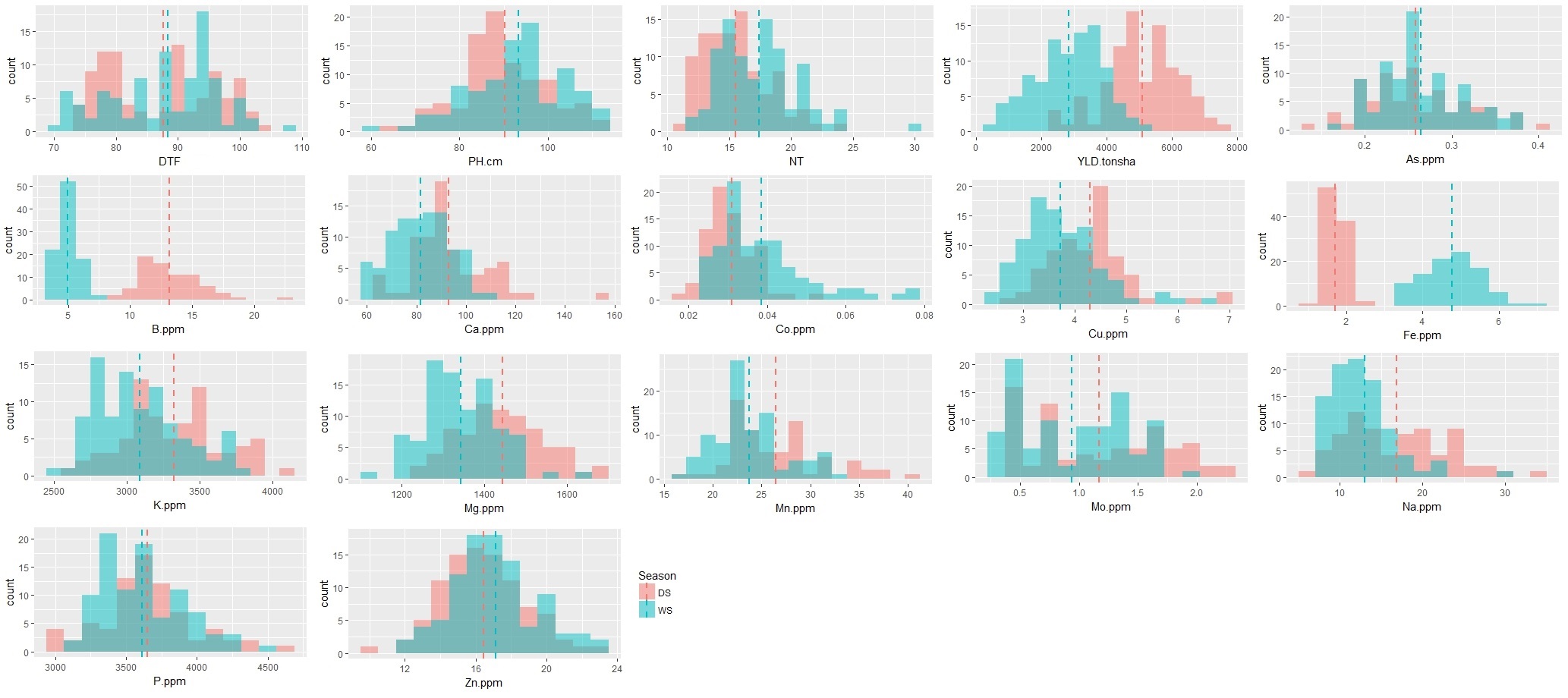

Supplement: S2 Fig — Red color: dry season (DS), green color: wet season (WS). (JPG) [file pone.0201756.s002.jpg]
